# Destroying glutathione peroxidase improves the oxidative stress resistance and pathogenicity of *Listeria monocytogenes*

**DOI:** 10.3389/fmicb.2023.1122623

**Published:** 2023-03-22

**Authors:** Yu Zhang, Qian Guo, Xiaowei Fang, Mei Yuan, Wenjie Hu, Xiongyan Liang, Jing Liu, Yuying Yang, Chun Fang

**Affiliations:** Laboratory of Animal Pathogenic Microbiology, College of Animal Science, Yangtze University, Jingzhou, China

**Keywords:** *Listeria monocytogenes*, glutathione peroxidase, glutathione, oxidative stress, virulence factor, pathogenicity

## Abstract

**Introduction:**

Glutathione peroxidase is abundant in eukaryotes as an important antioxidant enzyme. However, prokaryotic glutathione peroxidase has not been thoroughly studied. *Listeria monocytogenes* is a facultative intracellular pathogen that is capable of causing listeriosis in animals as well as humans. Despite the fact that *L. monocytogenes* encodes a putative glutathione peroxidase, GSH-Px (encoded by *lmo0983*)), the functions of the enzyme are still unknown. Here we revealed the unusual roles of *L. monocytogenes* GSH-Px in bacterial antioxidants and pathogenicity.

**Methods:**

*L. monocytogenes* Lm850658 was taken as the parental strain to construct the gsh-px deletion strain and related complement strain. The effect of the gsh-px gene on the resistance of *L. monocytogenes* to oxidative stress was determined by measuring the concentrations of glutathione and assaying the stress survival rates under different oxidative conditions. In addition, the pathogenicity of *L. monocytogenes* was determined by cellular adhesion and invasion assays and mice virulence tests, and the expression of virulence factors was determined by Western blot.

**Results:**

Deficiency of GSH-Px not only increased glutathione concentrations in L. monocytogenes but also enhanced its resistance to oxidative stress when exposed to copper and iron ions. In addition, the absence of gsh-px significantly improved the adhesion and invasion efficiency of *L. monocytogenes* to Caco-2 cells. More importantly, *L. monocytogenes* lacking GSH-Px could colonize and proliferate more efficiently in mice livers and spleens, enhancing the pathogenicity of L. monocytogenes by increasing the expression of virulence factors like InlA, InlB, and LLO.

**Discussion:**

Taken together, we confirmed that GSH-Px of *L. monocytogenes* has a counter-intuitive effect on the antioxidant capacity and pathogenicity.

## 1. Introduction

*Listeria monocytogenes* is found in plenty of environments and can easily transition from saprophytic organism to intracellular pathogen (Freitag et al., 2009; Xayarath and Freitag, 2012). Humans and animals may develop severe illnesses when they consume contaminated foods containing *L. monocytogenes* (Hedberg, 2011). *L. monocytogenes* faces a variety of stresses to survive in different niches, the most prevalent of which is oxidative stress. The oxidative conditions *L. monocytogenes* confronted include reactive oxygen species (ROS), reactive nitrogen species (RNS), and reactive chlorine species (RCS). Cellular components including DNA, proteins and membrane lipids are damaged when cells exposed to ROS, RNS, and RCS (Chakraborty et al., 1992; Ezraty et al., 2017). Consequently, for *L. monocytogenes* to thrive in oxidative environments, it needs to use a sophisticated system to ward off oxidative stress as well as prevent oxidative damage (Mates et al., 1999; Mates, 2000). To combat oxidative stress, *L. monocytogenes* encodes multiple metabolic enzymes like superoxide dismutase (SOD), catalase (CAT), thioredoxin (TRx), glutathione reductase (GR), and glutathione S-transferase (GST; Mains et al., 2021).

Glutathione peroxidases (GSH-Pxs) are a group of antioxidant enzymes found in eukaryotic organisms. In addition to being a vital component of the antioxidant systems, they may be involved in immune defense against pathogen invasion (Bathige et al., 2015). For example, inflammatory signaling cascades are closely linked to GSH-Px1 in mammals (Duong et al., 2010; Brigelius-Flohe and Maiorino, 2013). However, prokaryotic GSH-Px research is lacking. Numerous prokaryotes, including *Staphylococcus aureus*, *Bacillus subtilis*, *Lactococcus lactis*, and *L. monocytogenes*, have been found to possess genes homologous to *gsh-px* (Brenot et al., 2004). To date, there have been no investigations into the biological mechanisms of GSH-Px in bacteria in response to oxidative stress and host infection. GSH-Px reduces the organic hydroperoxides and hydrogen peroxide to the corresponding alcohols (water in the case of hydrogen peroxide) in eukaryotic organisms by utilizing glutathione as an electron donor (Ursini et al., 1995; Kutlu and Susuz, 2004). Whether GSH-Px in prokaryotes is closely linked to glutathione and affects oxidative resistance and pathogenicity, what specific biological functions of GSH-Px in prokaryotes are prompting us to understand the effects of GSH-Px on *L. monocytogenes*.

Glutathione (GSH), one of the glutaredoxin (Grx) systems, is necessary for preserving intracellular redox homeostasis (Masip et al., 2006). *L. monocytogenes* is one of the Gram-positive bacteria capable of synthesizing glutathione (Newton et al., 1996). GSH binds to PrfA at the broad tunnel between the N-terminal and C-terminal domains to make PrfA change conformation and become constitutive activation (Hall et al., 2016). PrfA is a global virulence regulator, which belongs to the Crp/Fnr transcriptional factor family (Scortti et al., 2007). GSH allosterically binds to PrfA results in activation of PrfA and exhibits high affinity to the promoters of PrfA regulated virulent genes (Chakraborty et al., 1992; Freitag et al., 2009; De Las Heras et al., 2011). Therefore, GSH improves the pathogenicity of *L. monocytogenes* by increasing the expression levels of PrfA regulated genes (Reniere et al., 2015; Hall et al., 2016). Moreover, it has been demonstrated that *L. monocytogenes* controlled its biphasic life cycle and transitioned from saprophytic to pathogenic by regulating the concentrations of GSH (Reniere et al., 2015). Hence GSH is indispensable for *L. monocytogenes* not only for its allosteric binding to PrfA to promote expression of the virulence factors but for the ability to resist oxidative stress to produce a reductive environment for life activity (Masip et al., 2006; Ku and Gan, 2019).

*Listeria monocytogenes lmo0983* has been identified as a putative glutathione peroxidase in the GenBank database. The purpose of the research is to confirm whether GSH-Px in *L. monocytogenes* is involved in the biological process of bacterial infection and the related molecular mechanisms. As shown by our findings that GSH-Px played unanticipated functions in the antioxidant and pathogenicity of *L. monocytogenes*. The resistance of *L. monocytogenes* to the oxidative condition induced by metal ions was significantly enhanced when the *gsh-px* gene has been deleted, and the virulence of the pathogen in cells and mice was also increased. The findings of this research provide valuable evidence for elucidating the multiple physiological roles of glutathione peroxidase in foodborne pathogens to survive in the external environment and, more importantly, in the successful establishment of infection in the host.

## 2. Materials and methods

### 2.1. Bacterial strains and culture conditions

*Listeria monocytogenes* Lm850658 was taken as a wild-type strain, and the other strains were derived from it. All *L. monocytogenes* strains were incubated in brain-heart infusion (BHI) medium (Oxoid). *E. coli* DH5α was utilized as a plasmid host strain and cultured in Luria-Bertani broth (LB) (Oxoid). Agar was added to solid media at 1% (w/v). All bacteria were incubated at 37°C unless otherwise requested. The following concentrations were used for the antibiotics: ampicillin (100 μg/ml), erythromycin (10 μg/ml), kanamycin (50 μg/ml), or chloramphenicol (10 μg/ml) were added appropriately to the medium. Sangon Biotech supplied the highest purity of all chemicals. Table 1 displays the primers utilized in the research.

**Table 1:** The PCR primers used in this study.

Primers	Sequeces(5′-3′)	Products(bp)
Δ*gsh-px*-A	CGC*GGATCC*ATAGAAGTAGGCGATTTTGTTTC	566
Δ*gsh-px*-B	GATGATGTCACTACCGAGTCCTTCATG*GAATTC*CTCCTATTTCAATATA	
Δ*gsh-px*-C	GAAGGACTCGGTAGTGACATCATC	563
Δ*gsh-px*-D	CGGGGTACCATTTACGCTTCCCTCCCATGTTTAA	
Δ*gsh-px*-AF	CGCGGATCCTGGTTATGGTGGTAATTCC	
CΔ*gsh-px*-F	TCC*CCCGGG*ATGAATGTCCATGATTTTTC	468
CΔ*gsh-px*-R	TTCGTCGACTTATTTACTTACTTTCGCAAC	

The restriction enzyme sites in Table 1 are not underlined.

### 2.2. In-frame deletion and complementation of *Listeria monocytogenes*

Construction of gene deletion strain utilizing a homologous recombination strategy (Camilli et al., 1993), and temperature-sensitive plasmid pKSV7 was employed as a shuttle vector. Briefly, PCR amplification yielded the upstream and downstream homologous arms of *lmo0983*. A knock-out plasmid was constructed by connecting the homologous arms to shuttle vector. To promote chromosomal integration, electroporation of the plasmid into Lm850658, and transformants were grown in BHI broth containing 10 μg/ml chloramphenicol at a non-permissive temperature of 42°C. In order to make plasmid excision and curing, the recombinants were continuously passaged without chloramphenicol at a permissive temperature of 30°C. Finally, the chloramphenicol-sensitive recombinant colonies were identified using PCR. The integrative plasmid pIMK2 was utilized to complement the *gsh-px* gene of *L. monocytogenes* (Monk et al., 2008). Briefly, the *gsh-px* gene and its endogenous promoter were obtained using the primer pairs shown in Table 1, which were cloned into the pIMK2 plasmid to create the complementation plasmid. Electroporation of complementary plasmid into *L. monocytogenes* Δ*gsh-px*. The constructed complement strain was named CΔ*gsh-px*.

### 2.3. Measurement of growth curves

All strains were streaked in advance on BHI plates and individual colonies of each strain were picked into 3 ml broth. All *L. monocytogenes* strains were incubated for an entire night at 37°C in BHI broth. Cultures were gathered by centrifugation at 12,000 rpm for 2 min and washed once in phosphate-buffered saline (PBS, 10 mM, pH 7.4). The optical density of the bacteria was standardized to 1.0. Bacterial cultures were diluted at a ratio of 1:100 with normal BHI broth or with H_2_O_2_ at sub-lethal concentrations of 10 mM. The diluted bacterial solution was added to 96-well plates at 200 μl per well, 3 parallel wells for each sample, and blank reference using BHI medium. All bacteria were cultivated at 37°C for 12 h. The kinetic growth was conducted and bacterial OD_600 nm_ was recorded at 1-h interval by a microplate reader.

### 2.4. Oxidative stress tests

H_2_O_2_ acted as a direct oxidant in oxidative stress, and the metal ions copper (Cu^2+^) and iron ions (Fe^3+^) acted as redox active stress (Birben et al., 2012). The overnight cultured *L. monocytogenes* was harvested by centrifuging at 12000 rpm for 2 min and washed once with PBS, then diluted to OD_600 nm_ of 1.0 (~10^9^ CFU/ml). The bacterial suspension was serially diluted after being resuspended in sterile PBS, and 100 μl bacterial solution was taken into 900 μl BHI broth containing 10 mM H_2_O_2_, 10 mM FeCl_3_, and 5 mM CuSO_4_ for 1 h, respectively. After incubating at 37°C for 1 h, the mixtures were serially diluted and placed on BHI agar plates. Spot plate counts were used to count the number of bacteria after incubation at 37°C for 24 h.

### 2.5. Determination of glutathione

GSH (reduced glutathione) concentrations were detected under different oxidative conditions. Beyotime Biotechnology provided the commercial measurement kits, which were used in accordance with the manufacturer’s instructions. In short, dilution of 10 mM GSSG (oxidized glutathione) stock solution with protein removal reagent M to 15, 10, 5, 2, 1, 0.5 μM sequentially, then the 6 points were taken to make a standard curve. Determination of bacterial samples needed to be performed on the basis of the fresh cells. Bacteria growing to the exponential stage were obtained by centrifugation at 12000 rpm for 5 min, which was weighed and treated with BHI broth containing 10 mM H_2_O_2_, 10 mM FeCl_3_, and 5 mM CuSO_4_ for 3 h, respectively. An equal amount of bacterial pellet was collected from the control group. All samples were treated with lysozyme at 37°C for 30 min, then added with protein removal reagent M 3 times the volume of the cell pellet and mixed thoroughly. The samples were rapidly freeze-thawed twice using liquid nitrogen and a 37°C water bath. The supernatant was gathered by centrifuging at 4°C and 12,000 rpm for 10 min, then the total glutathione concentrations could be determined. Some samples of total glutathione contents to be measured were taken. Adding diluted GSH removal solution proportionally with vortex immediately, then adding GSH scavenging reagent working solution. Immediately vortexed and reacted at 25°C for 60 min. The above reaction could remove GSH from the sample and be used to determine GSSG. The amount of GSH could be calculated by subtracting the amount of GSSG from the total glutathione (GSSG + GSH). GSH concentrations of the parental and mutant strains were measured as described above (Anaya-Sanchez et al., 2021). A 96-well plate was used for measurement, and the standards and samples were added in sequence. 150 μl total glutathione detection working solution was added to the wells, and 50 μl of 0.5 mg/ml NADPH solution was added after incubation at 25°C for 5 min. The absorbance of 412 nm was determined with a plate reader, measured every 10 min for 1 h.

### 2.6. Adhesion and invasion tests

The human intestinal epithelial Caco-2 cells were used for adhesion and invasion assays (Cheng et al., 2017b). In brief, the Caco-2 cells were cultured in Dulbecco’s modified Eagle’s medium (DMEM) supplemented with 10% fetal bovine serum (FBS) at 37°C with 5% CO_2_. Overnight grown *L. monocytogenes* was washed and resuspended with PBS. A single layer cells infected by bacteria for 30 min at a MOI of 10. For the adhesion assays, cells were lysed after two washes with PBS. As for the invasion assays, the prepared bacterial solution was added to the cells at 37°C with 5% CO_2_ for 1 h. To kill extracellular bacteria, DMEM comprising 200 mg/ml gentamicin was used for an additional 1 h after being washed twice with PBS. The cells were lysed after washing twice with PBS. Counting viable bacteria on BHI agar plates after the solution was 10-fold diluted. The adhesion or invasion efficiency was determined by dividing the number of colony-forming units (CFUs) of adherent or invasive cells by the total number of infected bacteria.

### 2.7. Virulence experiments

The virulence of *L. monocytogenes* parental and mutant strains was detected by the method of bacterial load in mice organs (livers, spleens, and brains; Cheng et al., 2017b). For the bacterial load, overnight-grown *L. monocytogenes* was diluted to concentrations of 1 × 10^7^ CFU/ml after washing once with PBS. The female Kunming mice were divided into 3 groups and 5 mice in each group. 10^6^ CFU of each strain were injected intraperitoneally into mice. The mice were euthanized 48 h post infection (pi), and samples of their brains, livers, and spleens were taken and homogenized in 1 ml PBS under sterile condition. In order to count the number of bacteria, after being serially diluted, the homogenate was placed on BHI agar plates and incubated overnight.

### 2.8. Western blot

*Listeria monocytogenes* uses a collection of virulence factors to increase its pathogenicity and establish an infection successfully, allosteric binding of GSH to the transcription regulator PrfA is necessary for the expression of the PrfA regulated virulence factors (Ku and Gan, 2019). Therefore, we conjectured that the deletion of *gsh-px* would affect the expression of virulence factors in *L. monocytogenes*. Specifically, the bacteria colonies of the parental strain and mutant strain were picked into 50 ml medium, respectively. Incubation in a shaker at 37°C for one night. For lysates of whole cells (Lenz and Portnoy, 2002), the bacteria was gathered by centrifugation at 12000 rpm for 10 min, bacterial pellet was resuspended with 500 μl extraction solution (2% SDS, 4% triton, 10 mM PBS). The mixture was freeze-thawed twice at −20°C for 1 h and 37°C for 15 min, the supernatant was obtained by centrifugation at 12000 rpm for 10 min as the whole cell extract. After being boiled for 10 min, the protein samples were isolated by SDS-PAGE and incubated with α-InlA, InlB, LLO, and α-GAPDH antisera (prepared in this study). GAPDH served as an internal reference for proteins extracted from whole cells. Referring to the former study, all primary antibodies were produced in our laboratory (Cheng et al., 2017a). Rabbits were injected subcutaneously with 500 μg protein, including InlA, InlB, LLO, and GAPDH separately, with an equivalent volume of complete adjuvant for primary immunization. After 14 days, each rabbit was injected subcutaneously with 250 μg of incomplete adjuvant and an equal volume of protein, the immunization was given three times, once every 2 weeks. Blood was obtained from rabbits 7 days after the last immunization. Primary antibodies were diluted at 1:5,00 – 5,000, including rabbit polyclonal antibodies against InlA, InlB, LLO, and GAPDH, and following the manufacturer’s instructions when using the appropriate secondary antibody. The enhanced chemiluminescence detection system was used to visualize all immunoreactions.

### 2.9. Ethics statement

Animal experiments had approved by the Laboratory Animal Management Committee of Yangtze University (Approval No. 20210301).

### 2.10. Statistical analysis

All experiments were performed three times with biological replicates. Data was expressed as mean ± standard deviation and statistical analysis was performed with GraphPad Prism 6 (version 6, GraphPad, United States). Unpaired t-tests were used to analyze the statistical significance. *p*-values <0.05 or 0.01 were considered statistically significant difference and marked with “*” and “**”; *p*-values >0.05 were considered as statistically, no significant difference and marked with “ns”.

## 3. Results

### 3.1. *In vitro* growth of *Listeria monocytogenes* was unaffected by the deletion of *gsh-px*

**Figure 1:**
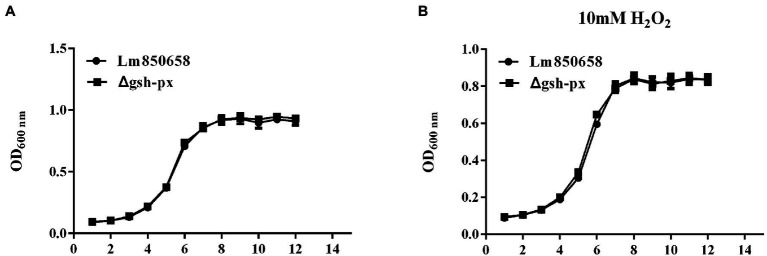
Deletion of *gsh-px* did no effect on the growth of *L. monocytogenes in vitro*. Overnight-grown bacteria were washed and diluted (1:100) in fresh BHI broth with **(B)** or without H_2_O_2_ **(A)**, incubated at 37°C for 12 h. Data were expressed as mean ± SD.

GSH-Px is a vital component of the antioxidant systems in eukaryotes (Margis et al., 2008). To learn more about the specific biological roles of GSH-Px in *L. monocytogenes*, in-frame deletion mutant strain of the *gsh-px* gene was constructed. To understand whether the deletion of *gsh-px* was relevant for the growth of *L. monocytogenes*, the growth capacity of parental strain Lm850658 and the deletion strain Δ*gsh-px* in BHI with or without 10 mM H_2_O_2_ was determined. After incubating all strains at 37°C for 12 h, it was found that the growth ability of the parental and deletion strains was comparable under the normal condition (Figure 1A). When treated with 10 mM H_2_O_2_, this phenomenon remained unchanged (Figure 1B), indicating that the absence of *gsh-px* had no effect on H_2_O_2_. This observation demonstrated that the deletion of *gsh-px* had no influence on the growth of bacteria *in vitro*.

### 3.2. Deficiency of *gsh-px* dramatically increased tolerance to the copper and iron but not to the hydrogen peroxide

**Figure 2:**
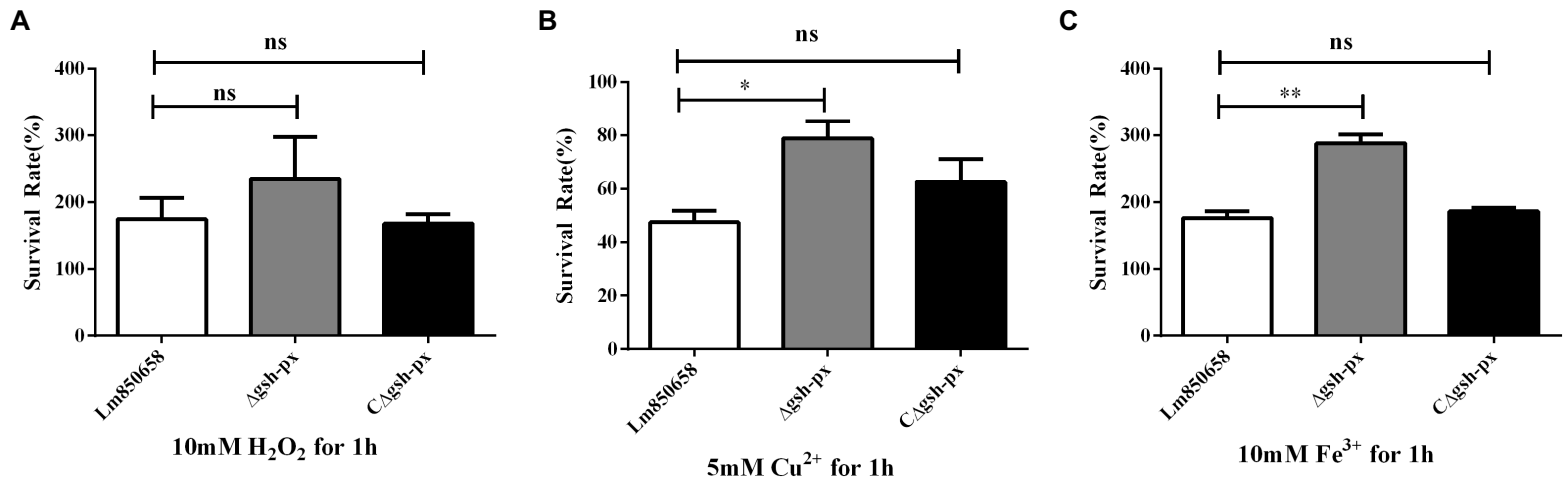
Deletion *gsh-px* of *L. monocytogenes* increased the tolerance to copper and iron, but not to hydrogen peroxide. Bacteria were treated with 10 mM H_2_O_2_ **(A)**, 5 mM Cu^2+^ **(B)** and 10 mM Fe^3+^ **(C)** for 1 h, and alive bacteria were counted by plate counting. The experiments were conducted for triplicate and data were expressed as mean ± SD. ns, no significance; *, *p* < 0.05; **, *p* < 0.01.

Since it has been shown that GSH-Px is closely related to the antioxidant systems of eukaryotes, further to identify whether GSH-Px of *L. monocytogenes* is correlated with bacterial oxidative resistance, the Lm850658 and *gsh-px* mutant strains (Δ*gsh-px* and CΔ*gsh-px*) were treated with diverse oxidizing media. By comparing the survival rates of the parental strain and deletional strain under the stress of the H_2_O_2_, the results showed that the survival rates of the parental strain and the mutant were comparable at 10 mM sub-lethal concentrations (Figure 2A). However, when exposed to the same concentrations of Fe^3+^, the survival rate of the deletion strain was significantly higher than that of the parental strain (Figure 2B), while no viable bacteria were detected in all strains at the same concentrations of Cu^2+^, so the concentrations of Cu^2+^ were reduced to 5 mM. The deletion of *gsh-px* significantly enhanced the resistance of *L. monocytogenes* to 5 mM Cu^2+^ (Figure 2C). This phenotype could be restored in the complementation strain CΔ*gsh-px*. These findings indicated that GSH-Px played an exceptional role in the antioxidant process of *L. monocytogenes* to metal ions, which was different from the classical fact that GSH-Px plays a role in enhancing antioxidant processes in eukaryotes.

### 3.3. The GSH concentrations of *Listeria monocytogenes* were significantly increased under the stimulation of metal ions

**Figure 3:**
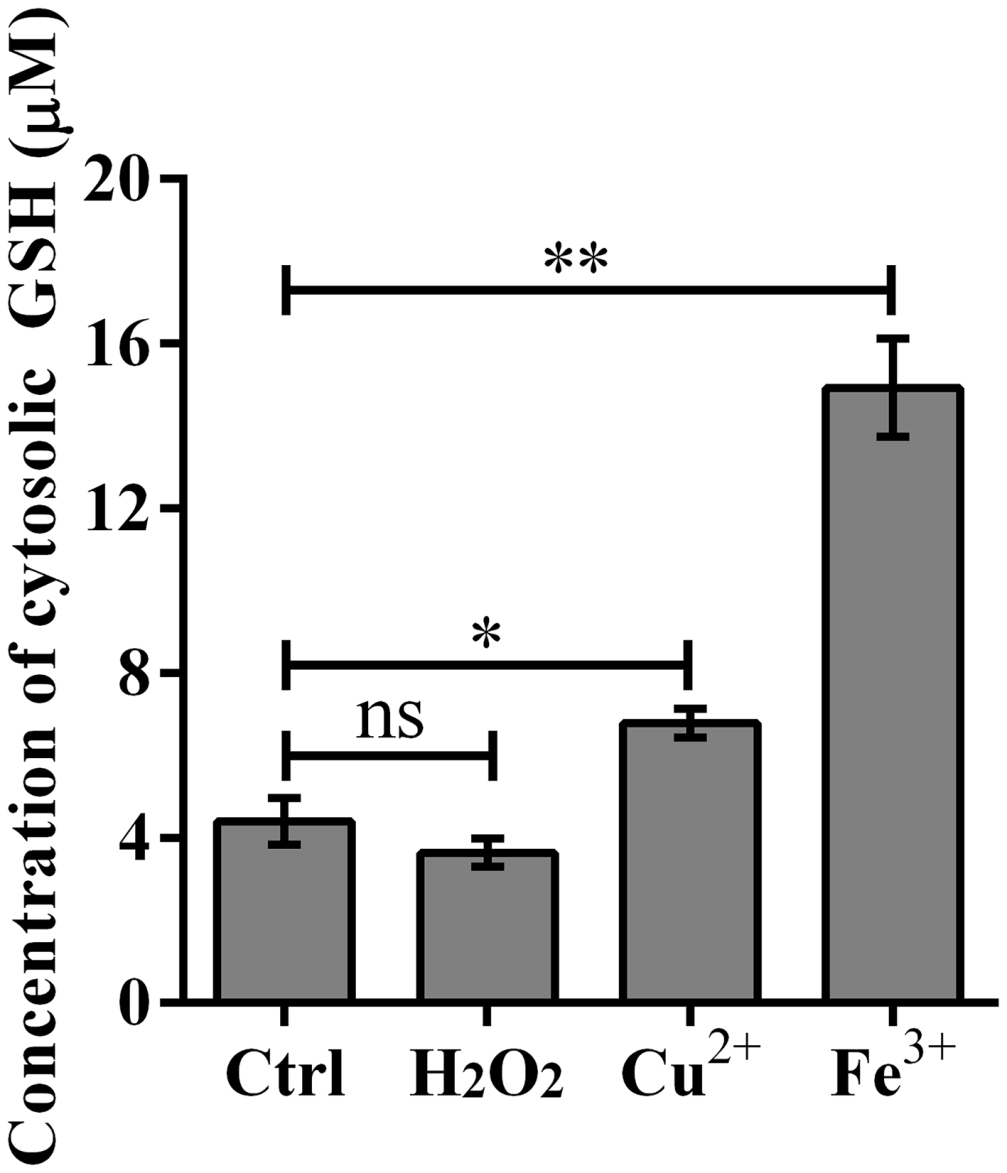
Cytosolic GSH concentration was significantly improved upon copper and iron treatment. Wild type strain Lm850658 was treated with BHI containing 10 mM hydrogen peroxide, 5 mM copper or 10 mM iron for 3 h and then bacteria were collected and lysed with lysozyme for 30 min. The concentrations of GSH were measured with commercial kit supplied by Beyotime Biotechnology as manufacturer’s instruction. The experiments were conducted for triplicate and data were expressed as mean ± SD. ns, no significance; *, *p* < 0.05; **, *p* < 0.01.

To understand whether the unusual effects of GSH-Px in *L. monocytogenes* are related to GSH, the concentrations of GSH under different oxidative conditions were measured. According to the measurement results, we found that in contrast to the normal BHI control group, the GSH of *L. monocytogenes* treated with metal ions for 3 h was significantly increased (Figure 3). However, after treating the bacteria with H_2_O_2_ for 3 h, GSH concentrations were essentially the same between the experimental and control groups (Figure 3). Unlike the role of GSH-Px in mammals, GSH concentrations in *L. monocytogenes* did not change significantly when H_2_O_2_ was used as a stressor, which was consistent with the results of oxidative stress tests.

### 3.4. Intracellular adhesion and invasion efficiency of *Listeria monocytogenes* was enhanced by the *gsh-px* mutation

**Figure 4:**
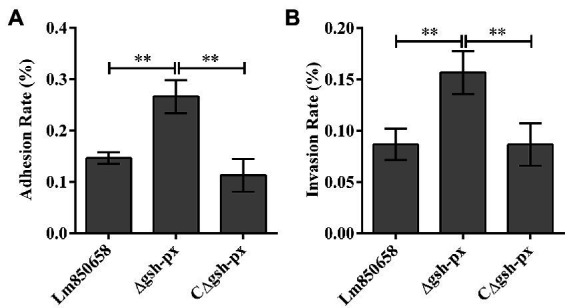
Mutation of *gsh-px* increased the adhesion **(A)** and invasion **(B)** ability of *L. monocytogenes* to Caco-2 cells. Epithelial cells Caco-2 were incubated with Lm850658, Δ*gsh-px* and CΔ*gsh-px* for 1 h, the adhesive bacteria were collected and counted by plate counting and the invasive bacteria collected after killing the extracellular bacteria with gentamycin. Finally, the adhesion and adhesion rates were calculated using the adherent and invasive bacteria divided by the initial bacteria. The experiments were conducted for triplicate and data were expressed as mean ± SD. ns, no significance; *, *p* < 0.05; **, *p* < 0.01.

Due to the unusual effect of GSH-Px on resistance to oxidative stress in *L. monocytogenes*, we set out to learn more about the role that GSH-Px plays in bacterial intracellular infection. Epithelial cell Caco-2 was used to compare the adhesion and invasion of wild-type and mutant strains. Surprisingly, the Δ*gsh-px* mutant strain was able to adhere and invade cells more effectively than parental strain (Figure 4), suggesting that the *gsh-px* gene was associated with the invasive ability of *L. monocytogenes* and may be redundant in helping the bacteria to invade the host.

### 3.5. Absence of *gsh-px* upgraded the harmfulness of *Listeria monocytogenes* to mice

**Figure 5:**
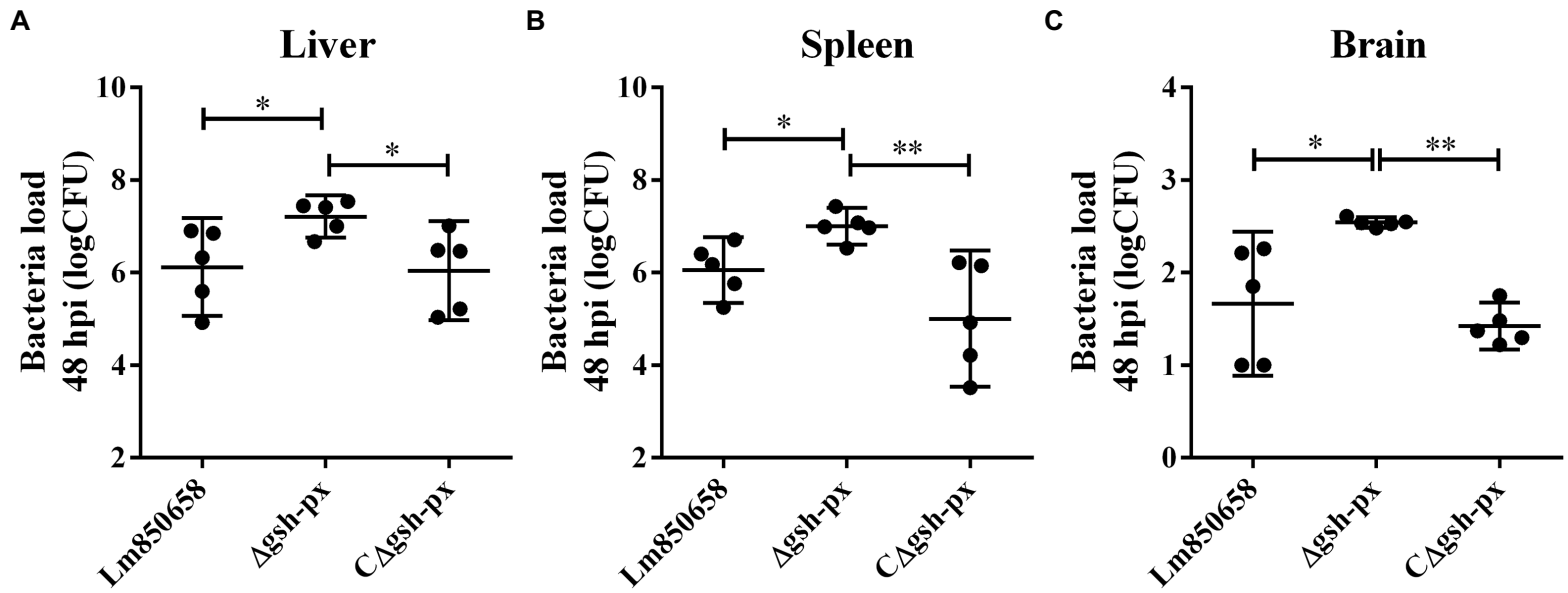
Deletion of *gsh-px* enhanced the virulence of *L. monocytogenes* to mice. Mice were infected with 10^7^ CFU by intraperitoneal injection with Lm850658, Δ*gsh-px* and CΔ*gsh-px*. Bacteria load in the livers **(A)**, spleens **(B)** and brains **(C)** were counted by plate counting after mice were euthanasia at 48 h post infection. Data were expressed as mean ± SD. ns, no significance; *, *p* < 0.05; **, *p* < 0.01.

The increased cell adhesion and invasion rates of the *gsh-px* mutant strain prompted us to explore more about how the GSH-Px affects virulence of *L. monocytogenes* in mammals. Therefore, we verified the connection between GSH-Px and virulence by measuring the bacterial load in the organs of mice. The results indicated that the Δ*gsh-px* strain recovered more CFU from the spleens, livers and brains of infected mice compared to the wild-type strain (Figure 5). According to the findings, enhanced virulence of the Δ*gsh-px* mutant strain in mice, which was consistent with the consequence of cellular assays. The remarkable role of GSH-Px in the pathogenicity of *L. monocytogenes* was demonstrated by the discovery of the *gsh-px* mutant that increased virulence.

### 3.6. Deletion of *gsh-px* upregulated the expression of major virulence factors in *Listeria monocytogenes*

**Figure 6:**
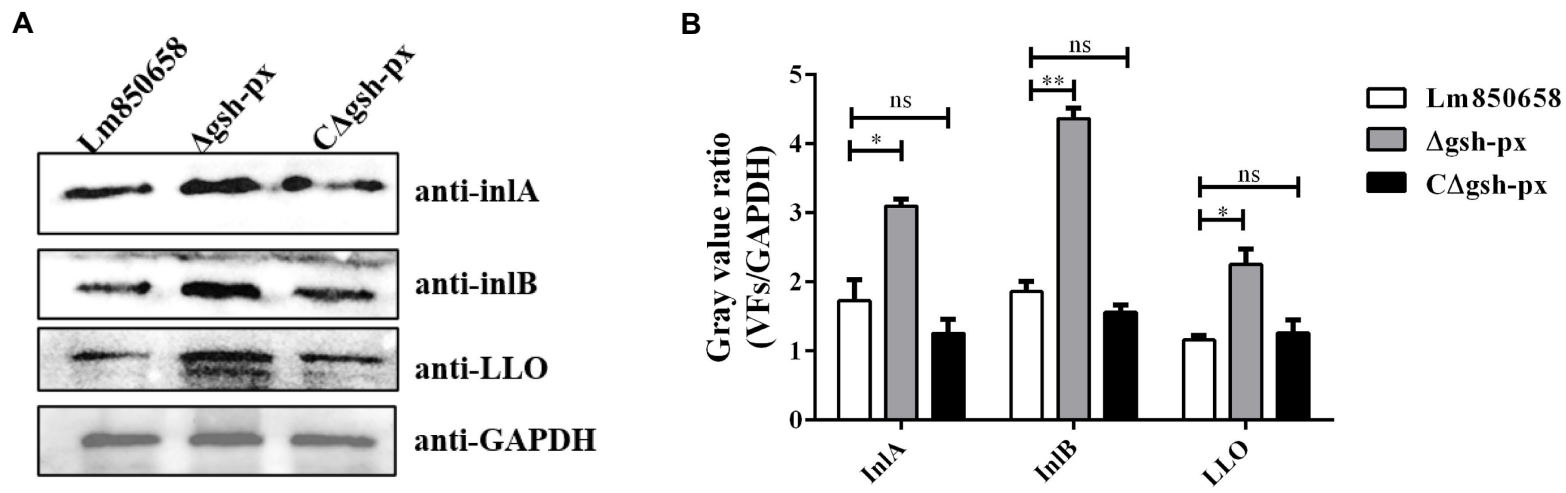
Deletion of *gsh-px* upregulated the expression of major virulence factors in *L. monocytogenes.* Overnight cultured bacteria were collected and the expression levels of the major virulence factor InlA, InlB and LLO were measured by western blot **(A)**. The gray value ratio was calculated by the band gray value of virulence factor relative to that of GAPDH **(B)**. Data were expressed as mean ± SD. ns, no significance; *, *p* < 0.05; **, *p* < 0.01.

The InlA and InlB are vital members of the virulence factors and the adhesion and invasion of *L. monocytogenes* into cells are crucially influenced by the InlA and InlB. By rupturing phagocytic vesicles, LLO makes it possible for bacteria to enter the cytoplasm. Western blotting was used to examine the expression of the major virulence factors in order to expand our understanding of how GSH-Px affects these factors. We discovered that *L. monocytogenes* was capable of invading the host more effectively in the absence of *gsh-px* in cellular experiments and virulence assays on mice. Based on this, we hypothesized that the increased expression of virulence factors might result from the deletion of *gsh-px*. The blotting validation results indicated that the expression of InlA, InlB and LLO in *L. monocytogenes* was remarkably increased when *gsh-px* was lacking, further to confirm the regulatory role of *gsh-px* on virulence factors in *L. monocytogenes* (Figure 6). This also supported our hypothesis that the deletion of *gsh-px* enhanced the adhesion and invasion ability of *L. monocytogenes* by enhancing the expression of virulence factors.

### 3.7. Deletion of *gsh-px* increased the GSH levels in *Listeria monocytogenes*

**Figure 7:**
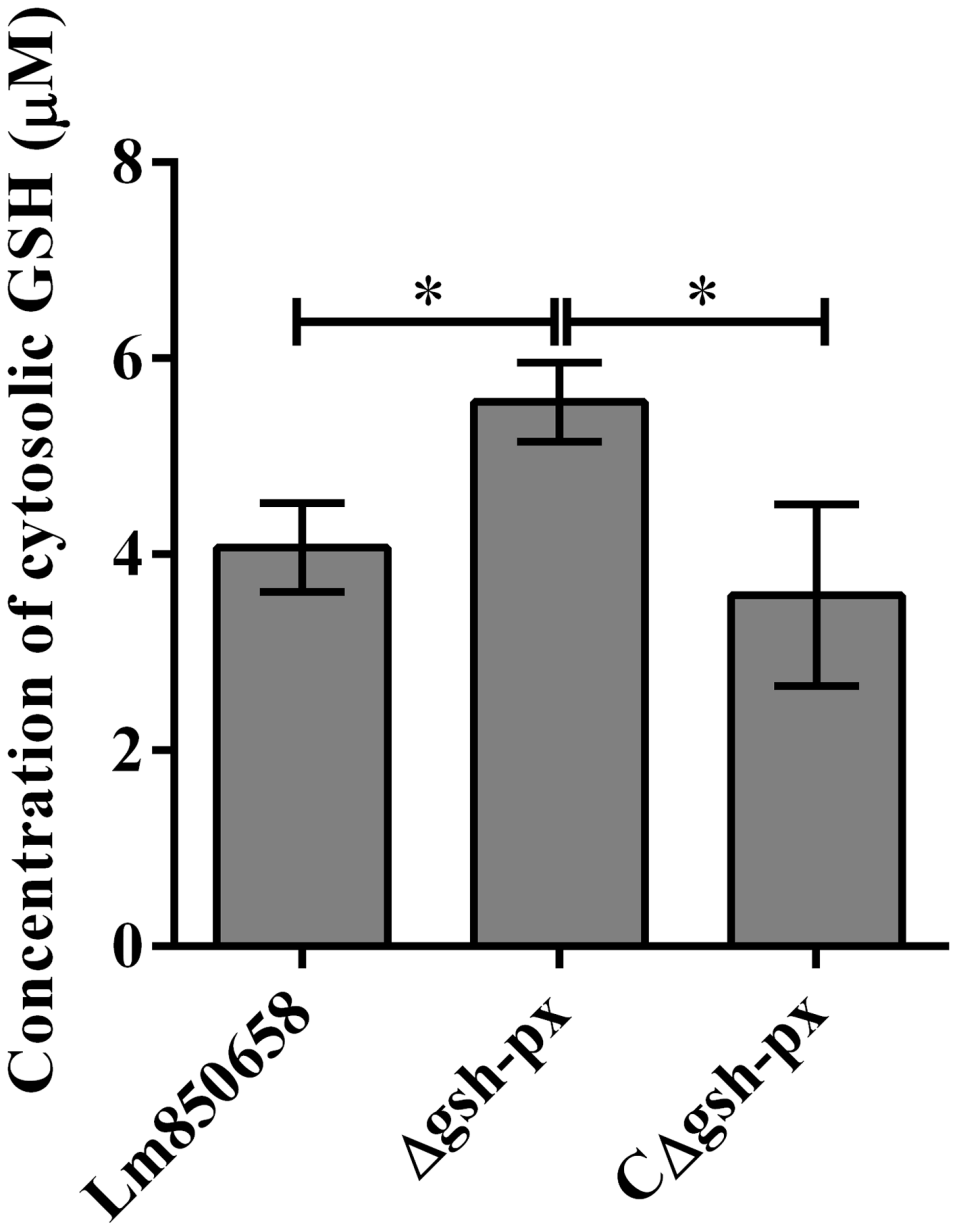
Deletion of *gsh-px* increased the glutathione levels in *L. monocytogenes*. Lm850658, Δ*gsh-px* and CΔ*gsh-px* were collected and lysed with lysozyme for 30 min. The concentrations of GSH were measured with commercial kit supplied by Beyotime Biotechnology as manufacturer’s instruction. The experiments were conducted for triplicate and data were expressed as mean ± SD. ns, no significance; *, *p* < 0.05; **, *p* < 0.01.

Due to the fact that intracellular GSH levels are required for PrfA activation, it is necessary to maintain high intracellular GSH concentrations for *L. monocytogenes*. We hypothesized that the effective intracellular GSH levels of the *gsh-px* deficient mutant were higher than that of the parental strain, resulting in enhanced virulence of the deletion strain. To validate the assumption, we tested the levels of GSH in the wild-type, Δ*gsh-px* and CΔ*gsh-px* strains. The results showed that the GSH concentrations of deletion strain were higher than that of parental strain, and this phenotype could be restored in the complementation strain CΔ*gsh-px* (Figure 7), suggesting that *L. monocytogenes* no longer consumed GSH or the rates of GSH consumption slowed down when the *gsh-px* was deleted, leading to GSH accumulation and enhanced virulence of *L. monocytogenes*.

## 4. Discussion

In normal circumstances, intracellular pro-oxidant agents and antioxidant stressors are in a dynamic equilibrium. However, when the host is infected with bacteria, host cells promote the synthesis of hydrogen peroxide, superoxide, and hydroxyl radicals to prevent bacterial invasion (Mates, 2000; van der Oost et al., 2003). The accumulation of pro-oxidant agents that cause oxidative damage in activated cells turns the cytoplasm into a risky location. Protein damage, oxidative degradation of lipids, DNA denaturation, apoptosis and necrosis are all outcomes of oxidative damage (Limon-Pacheco and Gonsebatt, 2009), because specialized phagocytes like macrophages can prevent pathogens from becoming viable through an oxidative burst of NADPH oxidase complexes assembled on phagocytic vesicles. Furthermore, some metals can also mediate the production of oxidative stimuli. In order to safeguard themselves from oxidative stressors and prevent essential virulent proteins from damaging, bacteria have naturally developed numerous antioxidant defense mechanisms, both enzymatic and non-enzymatic (Mates et al., 1999; Mates, 2000). As a foodborne pathogen, *L. monocytogenes* also contains several antioxidant enzymes to combat the strict conditions from both outside and inside environments of host. Superoxide dismutase (SOD) easily breaks down superoxide into peroxides, which indirectly causes the accumulation of H_2_O_2_ within the cell. Peroxides can be broken down by catalase (CAT) and peroxidase (PER), or the Fenton reaction with metal ions (Fe^2+^ and Cu^2+^) can turn them into hydroxyl radicals (Reichmann et al., 2018). However, the products of the Fenton reaction can also damage cells, through DNA or protein repair mechanisms, this can be reduced by removing or synthesizing new substances to replace the damaged cellular components.

By using GSH as an electron donor and catalyzing the conversion of organic hydroperoxides and hydrogen peroxide to the corresponding alcohols, GSH-Pxs in eukaryotes play a crucial role in mediating antioxidant defense (Arthur, 2000). However, prokaryotic GSH-Px is poorly understood, in contrast to the extensive research on eukaryotic GSH-Px. A previous report on the *gsh-px* gene in prokaryotes, *Neisseria meningitidis*, was published (Moore and Sparling, 1995, 1996). The susceptibility of *N. meningitidis* to oxidative stress caused by the redox cyclist paraquat was increased when the *gpxA* gene was inactivated (Moore and Sparling, 1996). Eukaryotic cells lacking GSH-Px activity experienced a similar phenomenon (Arthur, 2000). With the increased knowledge of prokaryotic GSH-Px, the *btuE* gene from *E. coli* and the *gpoA* gene from *Streptococcus pyogenes* are genes encoding various bacterial GSH-Px (Aho and Kelly, 1995; Moore and Sparling, 1996; King et al., 2000; Brenot et al., 2004). In *E. coli*, GSH-Px encoded by *btuE* suggests that this enzyme reduces cell oxidative stress and prevents cells from being damaged by a variety of oxidants (Arenas et al., 2010). Increased susceptibility of eukaryotic cells to paraquat is indeed present in *N. meningitidis gpoA* deletion mutant (Brenot et al., 2005), indicating that GSH-Px does aid in oxidative stress resistance. Surprisingly, in present research the tolerance of *L. monocytogenes* to metal ion-induced oxidative stress was significantly improved when *gsh-px* was not present, suggesting that GSH-Px has a remarkable effect in *L. monocytogenes*, contrary to the classical finding that the existence of GSH-Px in *E. coli* and *N. meningitidis* contributes to the antioxidant processes. However, the *gsh-px* deletion strain in *L. monocytogenes* was not affected by H_2_O_2_-induced oxidative stress. This may be due to additional antioxidant enzymes like CAT and SOD are present, which are so powerful and sensitive to H_2_O_2_-induced oxidative stress that GSH-Px did not respond significantly to it.

Audrey found that knock out *gsh-px* resulted in significantly impaired virulence of *S. pyogenes* in mice subcutaneous infection model, which provided the first concrete evidence that GSH-Px contributed to the pathogenicity of bacteria (Brenot et al., 2004, 2007). In the present work, we found enhanced virulence of *L. monocytogenes* when lacking the *gsh-px* gene, together with its adhesion and invasion efficiency, which could be accomplished by binding GSH to PrfA allosterically (Wang et al., 2017). GSH is a necessary small-molecule cofactor for PrfA, which is directly charged with the transcription of 10 essential virulence factors and has an indirect impact on the expression of over 140 other genes in *L. monocytogenes*. As a result, PrfA is regarded as the regulator of the major virulence factors, including *Listeria* pathogenic island 1 (LIPI-1) and LIPI-2, which contain *plcA-prfA-hly-mpl-actA-plcB* gene cluster and *inlA-inlB* gene cluster, respectively (De Las Heras et al., 2011). Due to the possibility of ROS, RNS and RCS being present in the phagocytic vesicles, glutathione dimers to form oxidized glutathione disulfide (GSSG), which does not bind to PrfA. However, since the host cytoplasm is a highly reduced state, once *L. monocytogenes* escape into the cytoplasm, all thiols are present in reduced form and the GSH is able to bind PrfA and trigger transcription of PrfA regulatory genes (PRG). Therefore, GSH acts as an irreplaceable element in the metabolic activation of PrfA (Reniere et al., 2015; Hall et al., 2016). Deletion of *gsh-px* gene increased concentrations of GSH in the mutant, which improved the efficiency of allosteric binding of PrfA to GSH and enhanced the regulation of virulence proteins like InlA, InlB and LLO (Kim et al., 2005), increasing pathogenicity of the *gsh-px* deletion strain. Moreover, transcriptome data analysis in a study revealed that the *lmo0983* (*gsh-px*) gene was apparently down-regulated in Δ*grx* strains of *L. monocytogenes* (Sun et al., 2019). The absence of *grx* contributed to improve survival of *L. monocytogenes* when exposed to copper and cadmium ions (Sun et al., 2019), it is speculated that the resistance to oxidative stress in *L. monocytogenes* should be up-regulated when *gsh-px* was absent, which was consistent with our experimental results.

The resistance of GSH-Px to oxidative stress is linked to virulence and other bacterial species as previously mentioned. We hypothesized that increased oxidative stress resistance might be the cause of an unexpected change in the pathogenicity of *gsh-px* mutants. This prompts us to consider how GSH-Px of *L. monocytogenes* perceives and reacts to the presence of metal ions. Perhaps conformation of GSH-Px is altered or the activity of GSH-Px is changed after stimulation by metal ions, which affects the expression of GSH-Px. Figuring out the mechanisms involved is the goal of our subsequent research. In conclusion, we provided worthwhile insight into a role of GSH-Px in bacterial infections by demonstrating the *L. monocytogenes* GSH-Px acts a counter-intuitive agent in bacterial oxidative tolerance and intracellular infection.

## Data availability statement

The original contributions presented in the study are included in the article/Supplementary material, further inquiries can be directed to the corresponding author.

## Ethics statement

The animal study was reviewed and approved by Animal Ethics Committee of Yangtze University.

## Author contributions

YZ and CF planned the experimental protocol and finished the manuscript. YZ and QG accomplished the main part of the study. XF, MY, and WH involved in the cell assay and animal assay. XL and JL participated in the gathering of experimental data. CF and YY participated in revising the manuscript. CF funded and oversaw the entire project. All authors contributed to the article and approved the submitted version.

## Funding

The work was sponsored by the National Natural Science Foundation of China (31802208) and the Science and Technology Research Project of Education Department of Hubei Province (Q20221302).

## Conflict of interest

The authors declare that the research was conducted in the absence of any commercial or financial relationships that could be construed as a potential conflict of interest.

## Publisher’s note

All claims expressed in this article are solely those of the authors and do not necessarily represent those of their affiliated organizations, or those of the publisher, the editors and the reviewers. Any product that may be evaluated in this article, or claim that may be made by its manufacturer, is not guaranteed or endorsed by the publisher.
